# Biochemical Responses of Medicinal Plant *Tussilago farfara* L. to Elevated Heavy Metal Concentrations in Soils of Urban Areas

**DOI:** 10.3390/toxics9070171

**Published:** 2021-07-17

**Authors:** Alexander Petukhov, Tatyana Kremleva, Galina Petukhova, Nikolay Khritokhin

**Affiliations:** Institute of Chemistry, University of Tyumen, 625003 Tyumen, Russia; t.a.kremleva@utmn.ru (T.K.); gpetuhova1@mail.ru (G.P.); kna@utmn.ru (N.K.)

**Keywords:** heavy metals, *Tussilago farfara* L., accumulation, urban area, antioxidants, lipid peroxidation

## Abstract

This study was conducted in Tyumen (Russian Federation) to establish the effects of heavy metals’ (Cu, Zn, Fe, Mn, Pb, and Cd) accumulation in soil and coltsfoot, as well as plants’ biochemical responses to such an accumulation. The mobile and acid-soluble heavy metal fractions in soils, and the heavy metal contents in plants, were determined by atomic absorption spectrophotometry. The Cu, Zn, Fe, Mn, and Pb concentrations in soils exceeded background values. Pb content at the battery manufacturing plant was above the maximum permitted concentration. The percentages of the mobile heavy metal fractions decreased in the following order: Mn > Zn > Cu > Fe. The greatest heavy metal accumulation in soils and plants was found at the battery manufacturing and metallurgical plants examined in our study. Heavy metals’ accumulation in the aboveground part of *Tussilago farfara* decreased in the following order: Fe > Zn > Cu > Mn > Pb > Cd. The accumulation of heavy metals stimulated the synthesis of photosynthetic pigments by 6–30%. Heavy metals provoked oxidative stress in cells, increasing the concentration of lipid peroxidation in products by up to 80%. Plant phenolics and flavonoids in the urban area of our study decreased compared to those in the control by 1.05, reaching up to 6.5 times. The change in coltsfoot catalase activity both increased and declined. Biochemical responses and heavy metal accumulation in coltsfoot from urban areas limit its use for medicinal purposes.

## 1. Introduction

The industrial development and intensification of agriculture has led to an increase in heavy metal concentrations in the environment. Due to their inability to biodegrade, their high toxicity, and an ability to affect food chain migration, heavy metals are only able to accumulate in ecosystems in increasing amounts, thus creating an ecological threat [1,2].

The sources of heavy metals in the environment may be effluents of metallurgical and mining facilities, industrial wastewaters, motor transport, combustion of fossil fuels, pesticides, and fertilizers [3,4]. The total anthropogenic emissions of several heavy metals in the atmosphere exceed those from natural sources by one to three orders of magnitude [5]. Independent of type and source of emission, heavy metals appear at the soil’s surface, which leads to their accumulation in the organogenic horizon and their absorbance by plant roots [6]. Metal accumulation in soil negatively affects the latter’s fertility, plant growth, and development. On average, approximately 11% of soils in the Russian Federation have a high level of heavy metal contamination [7].

During their evolution, plants have developed various adaptive mechanisms to counter increased concentrations of heavy metals in the environment. These include the binding of heavy metals by root exudates in the rhizosphere and cell wall, chelation in the cytoplasm, and isolation in vacuoles [8,9]. Only 0.2% percent of known plant species can accumulate heavy metals, and these have high metal concentrations above ground and a low content in the soil [9].

The toxic effects of heavy metals appear in their ability to bind with various functional groups in biomolecules, as well as their capacity to replace other ions in biomolecules, thus damaging their structure and activity [10,11]. Lipid peroxidation processes are also activated by heavy metals due to the electron transport inhibition of chloroplast and mitochondria membranes, the ability to generate free radicals from fatty acids, and the capacity to create reactive oxygen species (ROS) through Fenton and Haber–Weiss reactions [12,13].

Plants need antioxidants for limiting lipid peroxidation, which can terminate chains in radical oxidation, thus inactivating ROS and free radicals. Plant antioxidants are divided into nonenzymatic (carotenoids, plant phenolics, flavonoids, ascorbic acid, glutathione, and proline) and enzymatic (catalase, peroxidase, and superoxide dismutase) categories [14,15]. Antioxidant defense induction is carried out by energy provided from photosynthesis.

Coltsfoot (*Tussilago farfara* L., 1753) is a plant with widely acknowledged medicinal properties. Coltsfoot leaves possess expectorant, anti-inflammatory, disinfectant, and diaphoretic actions. Coltsfoot infusions are used for cough, bronchitis, laryngitis, bronchopneumonia, and bronchial asthma treatment [16]. *Tussilago farfara* is included in the pharmacopeias of 15 countries, and it is used in Russia, China, Turkey, Poland, the Balkan states, Germany, and other countries [17]. Coltsfoot is described in the literature as “nature’s best herb for the lungs” [18]. Research into the amino acid composition of coltsfoot leaves revealed a high arginine concentration, which positively effects the immune system [19]. All of this evidence for coltsfoots’ medicinal properties stipulates the relevance of this study in the context of rehabilitation after COVID-19 infection. Moreover, the chemistry of this plant has not been studied sufficiently [16].

Most of the studies in the literature are devoted to heavy metal concentrations in soils and plants of urban areas [20,21,22,23]. The complex investigation into plants’ biochemical status in relation to heavy metal accumulation in the environment remains urgent, as do determinations regarding the mechanisms that determine the effect of heavy metals on biochemical processes. This study was performed to investigate the effect of heavy metal concentrations on soils and plants in Tyumen (*Tussilago farfara* L.), as well as to determine the biochemical responses of plant cells.

## 2. Materials and Methods

### 2.1. Sampling Location

Tyumen (57°09′ N, 65°32′ E) is situated in Western Siberia and is the capital of the Tyumen Oblast in the Russian Federation, with a population of 800,000 people. Tyumen is a fast-developing center with high car traffic, developed oil and gas, battery-manufacturing, engine-building, and metallurgical industries. These factors can contribute to the potential soil pollution by heavy metals.

### 2.2. Sample Collection and Analysis

Samples were collected at the end of July 2019 at the following sites in Tyumen (Table 1).

Soils in the studied area were sod-podzolic. The area of all sites were no less than 100 square meters. Soils at 0–10 cm depth were collected at five spots for each site, averaged, and then transported to the laboratory in polyethylene bags. Air-dried soils were averaged by quartering, and were then ground and sifted through a 1 mm sieve. Some soil characteristics are presented in Table 2. Soils were close to neutral alkaline (pH = 6.6–8.3), had low humus content (0.5–1.5%), 52–409 mg kg^−1^ of K_2_O, 16–42 mg kg^−1^ ammoniacal nitrogen, 13–26 mg kg^−1^ nitrate nitrogen, and 17–238 mg kg^−1^ of mobile phosphorus.

Overground parts of coltsfoot (*Tussilago farfara* L., 1753) were collected at all corresponding examined sites. Plants were sampled with replicates (n = 100). Coltsfoot was one of the widespread plant species in the studied area, which stipulated the choice of sampling material. Our previous study indicated coltsfoot as most capable for heavy metal accumulation, at least compared to other herb species (Petukhov et al., 2020). The plants’ aboveground parts (all parts, including leaves and stems) were rinsed with deionized water, air-dried, and then ashed at 500 °C for 3 h. Plants and soils samples (for the determination of the acid-soluble heavy metal fraction) were digested with 5 M HNO_3_ for 3 h, and then filtered. The heavy metal mobile fraction content in soils was determined with ammonium acetate buffer (pH = 4.8) treatment for 24 h, and then samples were filtered. Heavy metal concentration was determined using a ContrAA 700 atomic absorption spectrophotometer (Analytic Jena, Jena, Germany).

### 2.3. Quality Control and Assurance

Solutions with standard metal concentrations were used to ensure the accuracy of the analytical procedure. Heavy metals recoveries ranged from 85% to 110%. All reagents were analytical reagent grade. All laboratory applications were conducted with double-distilled water.

### 2.4. Analysis of Biochemical Parameters

The study of chlorophyll a, b, and carotenoids was conducted by spectral analysis of alcohol extracts by reading absorbance at 662, 644, and 440 nm, respectively [24]. Diene conjugates and Schiff bases analysis was conducted in heptane extracts, and reading absorbance was at 233 nm and 365 nm, respectively [25]. Plant phenolic concentrations were determined by titration of plant aqueous extracts by potassium permanganate, until reaching a golden-yellow color, according to pharmacopeial standard [26]. Flavonoids’ determination was conducted in alcohol extracts by color reaction with aluminum chloride and consecutive absorbance reading at 410 nm [27]. Catalase activity analysis was performed by color reaction of hydrogen peroxide and ammonium molybdate; then, absorbance was read at 470 nm [28].

### 2.5. Statistical Analysis

The collected data were analyzed in the Statistica 10 software at the significance level *p* ≤ 0.05. Bivariate correlations were analyzed, and correlations were statistically significant if the correlation coefficient was R > 0.53 (*p* ≤ 0.05).

## 3. Results and Discussion

### 3.1. Characterization of Metal Content in Soils

The acid-soluble fraction of Cu in soils was higher than control by 1.1, and up to 2.8 times. The Fe acid-soluble form content exceeded the control level in almost all studied soils from Tyumen, especially at the BMP and UMMC (Table 3). Assumedly, the main Fe source in the environment is chimneys from the steel-making furnace at the metallurgical plant, as well as wastewater from lead–acid and iron–nickel battery production at the battery manufacturing plant. The concentration of mobile and acid-soluble Mn was elevated compared to control by up to 2.8-fold. This may be due to the appliance of Mn in antidetonant gasoline additives that are based on carbonyl manganese compounds, as well as the use of Mn in steel alloying. The concentration of Pb at the BMP exceeded control values, as well as the maximum permitted concentration in the Russian Federation (32 mg kg^−1^) [29]. This is likely attributable to lead–acid battery production. The Cd concentration in all soil samples was at the determination level (<0.34 mg kg^−1^). The mobile and acid-soluble forms of Zn were elevated by 1.1 and up to 6 times at all examined sites compared to control. The percentage of mobile fractions of heavy metals in the soil decreased in the following order: Mn > Zn > Cu > Fe. The highest concentrations of all studied metals were at the BMP and UMMC.

### 3.2. Heavy Metal Concentrations in Plants

**Figure 1:**
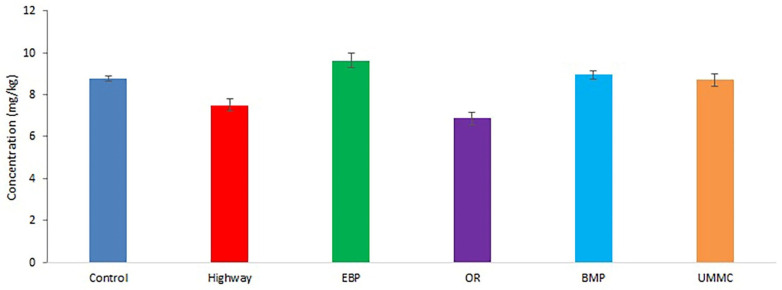
Cu concentration (mean ± 95% confidence interval) in coltsfoot from studied areas.

The Cu concentration in coltsfoot ranged from 7 to 10 mg kg^−1^ (Figure 1). The Cu content in plants was at the control level at all examined sites. This is considered to be the natural Cu content level in plants [30]. The maximum permitted level of Cu in forage for agricultural animals in the Russian Federation is 30 mg kg^−1^ [31]; there were no sites that exceeded this value. A similar result was found for Cu content in meadow grass at the Novocherkassk power plant [32]. The Cu content in plants was comparable to that in medicinal plants at a 7 km distance from the Middle Urals copper smelter [33], as well as with its content in herbs around former zinc smelters in the USA [34]. The Cu content in wild plants grown in contaminated soils in Southern Italy ranged from 4 to 31 mg kg^−1^, which is similar to the content this study [35]. Generally, similar Cu concentrations were found in herbs at former smelters in Slovenia [22].

**Figure 2:**
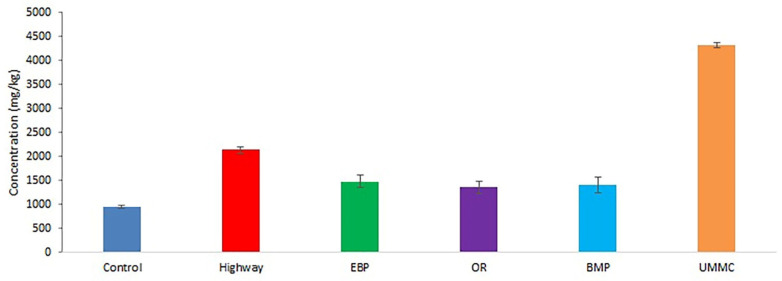
Fe concentration (mean ± 95% confidence interval) in coltsfoot from studied areas.

The Fe content in coltsfoot ranged from 941 to 4321 mg kg^−1^ (Figure 2). Similar Fe concentrations in herbs were found in Egypt [36] and Malaysia [37]. Fe content in plants from OR, EBP, and BMP was elevated by 40–55%. The highest Fe concentration was at the UMMC, exceeding the control level by 4.5 times. Presumably, chimneys of steelmaking production stipulate this result. High Fe concentration was also detected in plants near the highway, exceeding control more than two-fold. This may be due to the application of ferrocene and other Fe-containing antidetonant gasoline additives. Fe accumulation above 1000 mg kg^−1^ was earlier described in plants from Mn mining areas in China [38], as well as in technogenous areas of the Transbaikal region in Russia [39].

**Figure 3:**
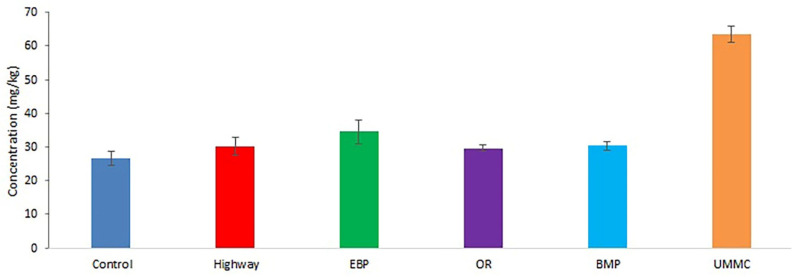
Mn concentration (mean ± 95% confidence interval) in coltsfoot from studied areas.

Mn concentration in coltsfoot ranged from 24 to 64 mg kg^−1^ (Figure 3). Mn content in plants at UMMC was elevated 2.4-fold compared to control. This is due to the use of Mn in steel alloying. Similar Mn concentrations were previously studied in plants from Novocherkassk [32] and Egypt [36].

Pb content in coltsfoot was at the determination level (5 mg kg^−1^), and no statistically significant differences were obtained. Similar Pb concentrations were registered in plants near the highway in Egypt [36], as well as in Malaysia [37].

Cd concentration in coltsfoot was at the determination level (0.34 mg kg^−1^). Similar Cd content was obtained in Novocherkassk [32], Malaysia [37], and Korea [20].

**Figure 4:**
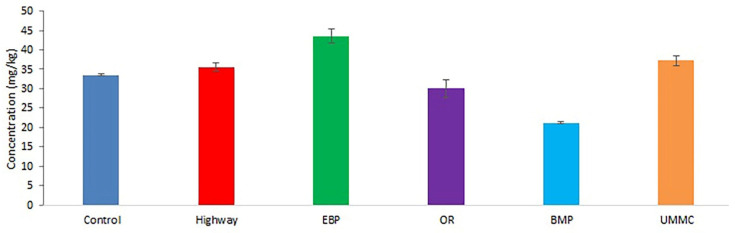
Zn concentration (mean ± 95% confidence interval) in coltsfoot from studied areas.

The Zn content in coltsfoot ranged from 21 to 44 mg kg^−1^ (Figure 4). Similar Zn concentrations were registered in most of the plants from a former mining area in Slovenia [22], as well as in a polluted area in the south of Italy [35]. Compared to control, elevated Zn content was found near the highway, the EBP, and the UMMC (by 25, 33, and 19%, respectively).

### 3.3. Analysis of Biochemical Parameters

**Figure 5:**
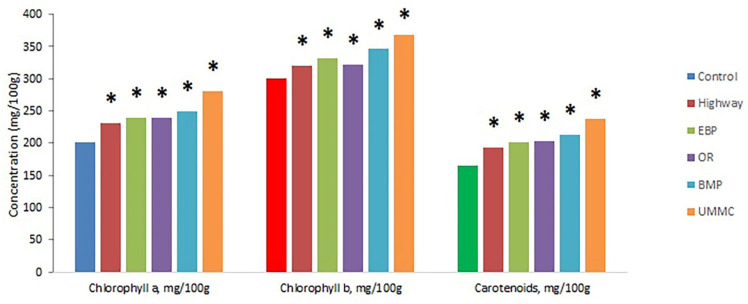
Concentration of photosynthetic pigments in coltsfoot from studied areas. *—statistically significant difference between control and test group (*p* ≤ 0.05).

Chlorophyll a, b, and carotenoid content in coltsfoot was higher at all examined sites compared to control (Figure 5). In most cases, this increment was 6–30%. The greatest increase was at the UMMC; chlorophyll a and carotenoid concentrations were elevated by 40–45%, while chlorophyll b was elevated by 20%. Such a reaction may be due to the need to synthesize antioxidant compounds in response to the stress of vegetation in urban areas (all soils except the control). This synthesis is provided by energy obtained in photosynthesis. That hypothesis is supported by the highest increase in chlorophyll content at the UMMC, where the greatest metal concentrations in both soils and plants were registered. An increase in photosynthetic pigment content under the effect of heavy metals was shown by Cd accumulation in *Lathyrus maritimus Bigel* [40], as well as the effects of Cu, Zn, Pb, and Cd on maize [41] and the treatment of wheat and mustard by wastewaters [42]. The rise in photosynthetic pigment concentrations may indicate a higher photosynthesis efficiency in urban area conditions [38,39,40]. This result is relevant due to climate change and the urgent need to curtail carbon dioxide emissions.

**Figure 6:**
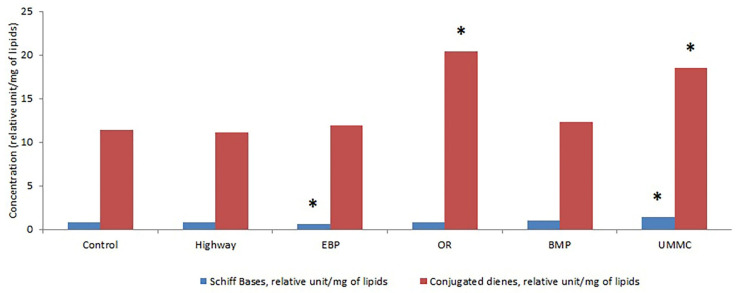
Concentration of lipid peroxidation products (Schiff bases and conjugated dienes) in coltsfoot from studied areas. *—statistically significant difference between control and test group (*p* ≤ 0.05).

The concentration of lipid peroxidation products in coltsfoot cells from the studied area mostly remained at the control level (Figure 6). Exceptions are a decrease in Schiff base concentrations at the EBP by 28% and an increase in diene conjugates at the OR and the UMMC by 80%, as well as a Schiff base increase at the UMMC by 67%. In conditions of heavy metal accumulation, antioxidants of both an enzymatic and a nonenzymatic nature may undergo disruption in their structures due to the binding of molecule functional groups and the replacement of essential ions with heavy metals. This can lead to an increase in the concentration of lipid peroxidation products, as is clear from our experiment. Due to high redox Fe^3+^/Fe^2+^ mobility, Fe can participate in lipid peroxidation, generating free radicals and reactive oxygen species by Fenton and Haber–Weiss reactions [13]. Fe accumulation in coltsfoot was the most intense at the UMMC, which in turn probably led to the highest development of oxidative processes in cells. Lipid peroxidation in plant cells under the effect of heavy metals was also detected in other studies [43,44,45,46,47]. Oxidative processes promote the generation of toxic compounds, as well as the decomposition of useful substances such as alkaloids, flavonoids, terpenoids, amino acids, and vitamins. This decreases the value of plants’ medicinal properties.

**Figure 7:**
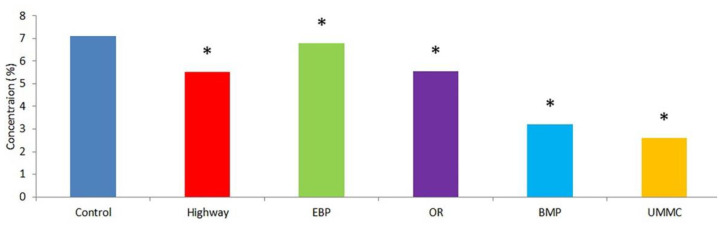
Plant phenolics concentration in coltsfoot from studied areas. *—statistically significant difference between control and test group (*p* ≤ 0.05).

The analysis of phenolic antioxidants in coltsfoot from the urban area in our study revealed an unambiguous tendency to decrease plant phenolics at all examined sites compared to control (Figure 7). Phenols content in coltsfoot decreased by 5% at the EBP and up to 65% at the UMMC. Notably, plant phenolics were affected the most at the UMMC, which was characterized by the highest heavy metal content in both soils and plants. This finding enables the use of phenolic antioxidants as ecological indicators of environmental conditions [40]. A decrease in plant phenolic content may be stipulated by its depletion to terminate lipid peroxidation free radical chains. Phenols themselves may be oxidized by ROS to quinone structures. Moreover, plant phenolics may chelate heavy metal ions, such as Cu^2+^, Zn^2+^, Fe^2+^, Fe^3+^, and Pb^2+^. A decrease in phenol compound concentrations in herbs in urban areas was shown earlier with the example of Kaliningrad [40].

**Figure 8:**
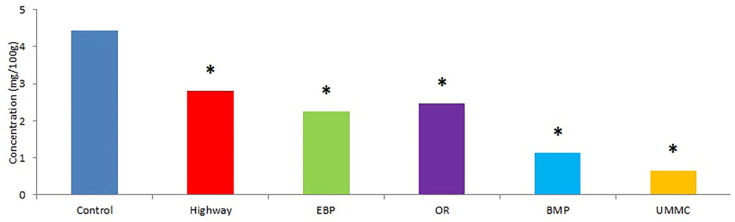
Flavonoids’ concentrations in coltsfoot from studied areas. *—statistically significant difference between control and test group (*p* ≤ 0.05).

Flavonoids’ content in coltsfoot from the urban area in our study was below the control level by 1.5 times, and up to 6.5 times, at all examined sites (Figure 8). Flavonoids are capable of producing donor protons and electrons, which terminate lipid peroxidation chains and decrease their detecting concentrations. Moreover, flavonoids can be substrates for various peroxidases by utilizing toxic hydrogen peroxide; this explains the reduction in their content. Environmental pollutants may oppress biosynthesis of phenolic antioxidants due to their damaging of enzyme structures (synthase, reductase) by ROS or the binding of heavy metals to –SH, –NH_2_, –OH_,_ –COOH functional groups. As is described in the literature, Cd accumulation decreased catechins and quercetin concentrations in mustard [48].

The phenolics and flavonoids of plants have antibacterial and anti-inflammatory effects, and are widely used in medicine. Decreasing concentrations of these substances in coltsfoot from urban area lowers this plant’s medicinal use.

**Figure 9:**
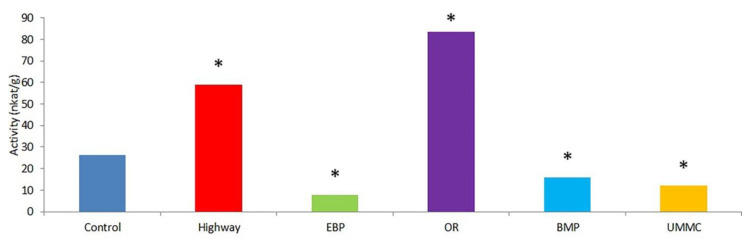
Catalase activity in coltsfoot from studied areas. *—statistically significant difference between control and test group (*p* ≤ 0.05).

Catalase activity in coltsfoot decreased by 40–70% at EBP, BMP, and UMMC (Figure 9). Meanwhile, enzyme activity at the OR and highway sharply increased. Catalase activation may be explained by changes in component composition. Enzymes in cells may be in a free or a bound state, while, in stress, the percentage of free forms increases due to the release of bound forms. A decrease in catalase activity can be provoked by damage in the enzyme structure or the oppression of catalase biosynthesis. Contaminants in plant cells can bind to –SH, –NH_2_, and –COOH groups of amino acids in the enzyme, as well as replace heme iron ions, which suppress enzymatic activity. It is understood that Cd negatively affects catalase activity in wheat seedlings, which also includes an increase in lipid peroxidation products [49]. A reduction in catalase activity in response to cadmium environmental pollution has also been described in watercress cells [50]. An ambiguous effect of heavy metals’ accumulation on catalase activity was detected in previous studies with model pollution experiments: Cu and Cd suppressed enzyme activity [50,51,52], while Pb, Ni, and Zn activated catalase [52,53,54]. A decrease in plant catalase activity also leads to the lower bioavailability of heme iron for humans, which negatively affects the medicinal properties of herbs.

### 3.4. Correlation Analysis

Heavy metal contents in soils positively correlated with one other: Cu–Fe (r = 0.96), Cu–Zn (r = 0.97), Mn–Fe (r = 0.61), Mn–Zn (r = 0.68), Fe–Zn (r = 0.92). These correlations indicate the complex character of heavy metal pollution in soil. Mn content in coltsfoot correlated with its mobile form in soil (r = 0.70). Fe and Mn concentrations in coltsfoot positively correlated with each other (r = 0.96).

Photosynthetic pigment concentrations in coltsfoot positively correlated with the heavy metal levels in soils and plants. For instance, Fe in plants correlated with chlorophyll a, b, and carotenoids (r = 0.82; 0.80; 0.78), as well as Mn (r = 0.83; 0.84; 0.80), acid-soluble Cu in soil (r = 0.65; 0.55), mobile Zn in soil (r = 0.90; 0.89; 0.87), and other metals in soil. Fe, Mn, Cu, and Zn are essential microelements for plants. Therefore, their absorbance can lead to an increase in photosynthesis pigments due to activation of chlorophyll biosynthesis enzymes, intensification of electron transport on photosynthetic membranes, and water photolysis. Heavy metals are known to stimulate all of these photosynthetic functions [2,7]. On the other hand, such correlations can mean metal stress for plants, which includes increasing demand for energy for phytochelatins and antioxidant synthesis. This hypothesis is supported by the correlation between chlorophyll a and b and Schiff bases (r = 0.70; 0.73).

Fe and Mn content in plants correlated with Schiff bases in cells (r = 0.82; 0.85). This may be due to the direct participation of these metals in lipid peroxidation, generating ROS and free radicals, as well as the indirect oppression of antioxidant functionality. Similar correlations were apparent for heavy metals in soils: mobile Cu in soil correlated with diene conjugates (r = 0.83), acid-soluble Mn and Schiff bases (r = 0.60), and mobile Zn and Schiff bases (r = 0.64). Such correlations indicate plant heavy metal stress both from their accumulation and from presence in the environment.

Plant phenolics negatively correlated with Fe and Mn in plants (r = −0.66; −0.70). This is apparent from the chelation of phenol hydroxyl groups in conditions of Fe and Mn accumulation in cells. Similar correlations were found for Cu, Zn, Fe, and Mn levels in soils and phenols, as well as flavonoid concentrations in plants. Phenolic antioxidants negatively correlated with Schiff bases (r = −0.84), which shows the successful antioxidative function of plant phenols. Meanwhile, carotenoids positively correlated with Schiff bases (r = 0.68). This is probably because of carotenoids’ synthesis due to their ability to deactivate superoxide anions.

Correlation analysis revealed a negative interconnection between Cu in plants and catalase activity (r = −0.97), as well as similar correlations for Cu, Mn, Zn, and Cd in soils (r = −0.60; −0.70; −0.73; −0.54). This is likely to be because of the damage to the enzyme’s structure and the replacement of heme iron with other metals.

## 4. Conclusions

1. Metal accumulation stimulated photosynthetic pigment synthesis in coltsfoot, while the metal content correlated with pigment concentrations. Heavy metals provoked oxidative stress in plants, which is registered by Fe and Mn correlations in plants with Schiff bases, as well as increments in lipid peroxidation products at the oil refinery and metallurgical plants. Phenols and flavonoids functioned as antioxidants and depleted for metal chelation. The heavy metal content of soils and plants was negatively correlated with catalase activity.
2. The heavy metal accumulation by plants in Tyumen decreased in the following order: Fe > Zn > Mn > Cu > Pb > Cd. Zn and Mn content in soils correlated with that in plants. The most intense metal accumulation was detected at a metallurgical plant.
3. The heavy metal analysis of soils revealed Fe contamination compared to control, as well as Pb pollution at the battery manufacturing plant. Furthermore, Cu, Mn, and Zn concentrations exceeded background levels at all examined sites. The percentage of heavy metals in mobile form decreased in the following order: Mn > Zn > Cu > Fe. The highest heavy metal concentrations in soil occurred at the battery manufacturing and metallurgical plants.
4. Coltsfoot vegetation in the polluted urban area and metal accumulation led to complex biochemical reactions. Coltsfoot’s biochemical responses and its ability to accumulate heavy metals limit the plant’s use for medicinal purposes.

## Figures and Tables

**Table 1:** Sampling locations.

No.	Name	Location
1	Control	Outside the city at 10 km distance from anthropogenic sources
2	Federal Highway Tyumen-Omsk	30 km from Tyumen, distance from the road no more than 30 m
3	Engine-building plant (EBP)	200 m from the plant
4	Oil refinery (OR)	200 m from Antipinsky Oil refinery
5	Battery Manufacturing plant (BMP)	200 m from the plant
6	UMMC (Ural Mining and Metallurgical Company)	200 m south of the enterprise

**Table 2:** Soil characteristics in the studied area.

	pH	Humus. %	K_2_O. mg kg^−1^	N(NH_4_^+^). mg kg^−1^	N(NO_3_^−^). mg kg^−1^	P_2_O_5_. mg kg^−1^
Control	6.8 ± 0.2	0.57 ± 0.06	313 ± 47	33 ± 15	26 ± 11	91 ± 18
Highway	6.6 ± 0.2	0.64 ± 0.06	85 ± 12	42 ± 19	16 ± 9	95 ± 19
EBP	7.8 ± 0.2	0.46 ± 0.04	409 ± 61	34 ± 16	13 ± 7	32 ± 6
OR	7.9 ± 0.2	0.58 ± 0.06	331 ± 50	37 ± 17	15 ± 8	135 ± 27
BMP	8.1 ± 0.2	1.53 ± 0.10	194 ± 29	23 ± 11	23 ± 12	17 ± 6
UMMC	8.3 ± 0.2	0.86 ± 0.08	52 ± 10	16 ± 6	20 ± 11	138 ± 28

**Table 3:** Heavy metal concentrations (mg kg^−1^ mean ± 95% confidence interval) in soils in Tyumen in 2019 (above the line—mobile forms; below the line—acid-soluble forms).

	Cu	Fe	Mn	Pb	Cd	Zn
Control	0.58 ± 0.15 5.93 ± 0.29	207 ± 9.68 28,500 ± 830	59.7 ± 6.03 171 ± 2.35	4.30 ± 2.19 7.34 ± 3.57	0.08 ± 0.03 0.33 ± 0.17	1.16 ± 0.05 16.5 ± 0.22
Highway	0.55 ± 0.20 3.69 ± 0.95	172 ± 13.5 25,700 ± 1100	71.0 ± 0.92 390 ± 7.72	4.35 ± 1.83 7.23 ± 3.73	0.13 ± 0.04 0.33 ± 0.16	3.62 ± 0.10 15.6 ± 0.51
EBP	0.50 ± 0.14 9.77 ± 0.46	67.8 ± 5.65 44,000 ± 1000	105 ± 1.56 276 ± 7.34	5.19 ± 1.76 8.14 ± 3.63	0.16 ± 0.08 0.34 ± 0.17	4.48 ± 0.16 28.0 ± 0.50
OR	0.72 ± 0.20 6.67 ± 0.33	102 ± 7.21 43,700 ± 1300	68.4 ± 0.88 282 ± 10.3	1.51 ± 0.87 7.29 ± 3.60	0.13 ± 0.03 0.33 ± 0.17	2.44 ± 0.16 19.2 ± 0.58
BMP	0.49 ± 0.09 16.6 ± 0.93	49.3 ± 7.45 95,000 ± 2300	96.5 ± 0.52 448 ± 12.0	34.9 ± 3.20 54.6 ± 3.70	0.13 ± 0.06 0.34 ± 0.17	3.20 ± 0.08 45.5 ± 1.32
UMMC	0.61 ± 0.20 10.8 ± 0.46	180 ± 5.02 53,500 ± 1200	110 ± 1.06 435 ± 10.6	4.89 ± 3.37 7.61 ± 3.73	0.14 ± 0.02 0.33 ± 0.17	7.28 ± 0.06 36.7 ± 2.19

## Data Availability

Data is contained within article.
